# O.1.1-6 Active in Mind: a 6-year synthesis of the effects of a physical activity and mental health-focused programme on young people in a school-based setting

**DOI:** 10.1093/eurpub/ckad133.082

**Published:** 2023-09-11

**Authors:** Alice Burgin, Lucy Slavin

**Affiliations:** Youth Sport Trust, UK[Corresp ckad133.082-cor1]alice.burgin@youthsporttrust.org; Youth Sport Trust, UK

## Abstract

**Purpose:**

Mental health issues in young people are rising and have many adverse impacts on their health, development and education. The Active in Mind programme is a free to access, school-based intervention that uses physical activity as a vehicle for improving young people’s physical, mental and social wellbeing, whilst addressing physical inactivity. The programme is led by an Athlete Mentor, alongside student mentors, and targets young people with low-moderate mental health issues, who are not receiving statutory support, as mentees.

**Project description:**

The Active in Mind programme was first piloted in 2016. Subsequent iterations of the programme have been based on evidence from both previous approaches of each implementation and evaluation, as well as the evolution of the needs of young people and schools. Together, the Active in Mind programme has always sought to address the current needs of schools and young people, with evidence-based core components that can be adapted based on school needs. To date, the programme has reached 21,216 young people. Survey data from young people at baseline and post-programme across this time period has been synthesised. Overall, synthesised outcome data from a subset of young people demonstrates improvements in mentees’ physical activity levels, mental and social wellbeing, whilst improving mentors’ life skills such as leadership, team working and communication skills. Differences in outcomes between mentors and mentees are present, as well as differences in young people from less and more affluent backgrounds.

**Conclusion:**

The current synthesis of self-reported data demonstrates the effectiveness of a free to access school-based physical activity programme on young people’s physical activity levels, mental and social wellbeing and life skills. A strength of the programme is its evidence-based implementation and evaluation and that it provides a solution for the current challenges schools are facing around young people’s mental and physical wellbeing.

The programme’s evaluations have contributed to design and development of future Youth Sport Trust programmes. In addition, evaluations of the programme have also evolved across the time period to ensure the most influential format of evidence is created, in order to influence external policymakers and decision makers at the time.

